# Silencing of *IRF8* Mediated by m6A Modification Promotes the Progression of T‐Cell Acute Lymphoblastic Leukemia

**DOI:** 10.1002/advs.202201724

**Published:** 2022-12-07

**Authors:** Ying Zhou, Min Ji, Yuan Xia, Xiaoyu Han, Mingying Li, Wei Li, Tao Sun, Jingru Zhang, Fei Lu, Yanping Sun, Na Liu, Jingxin Li, Daoxin Ma, Jingjing Ye, Chunyan Ji

**Affiliations:** ^1^ Department of Hematology Qilu Hospital Cheeloo College of Medicine Shandong University Jinan 250012 P. R. China; ^2^ Key Laboratory of Immunohematology Qilu Hospital Shandong University Jinan 250012 P. R. China; ^3^ Department of Physiology School of Basic Medical Sciences Cheeloo College of Medicine Shandong University Jinan 250012 P. R. China

**Keywords:** fat mass‐ and obesity‐associated protein (FTO), IRF8, m6A modification, PI3K/AKT signaling, PIK3R5

## Abstract

T‐cell acute lymphoblastic leukemia (T‐ALL) is an aggressive hematological malignancy with a poor prognosis, urging for novel therapeutic targets and treatment strategies. *N6*‐methyladenosine (m6A) is a crucial methylation modification that affects the pathogenesis of leukemia by regulating the mRNA of key genes. Interferon regulatory factor 8 (IRF8) is a crucial transcription factor for hematological lineage commitment, but its role in T‐ALL is unclear. Here, IRF8 is shown to suppress T‐ALL. The expression of IRF8 is abnormally silenced in patients with T‐ALL. Knockout of *Irf8* significantly hastens the progression of Notch1‐induced T‐ALL in vivo. Overexpression of IRF8 suppresses the proliferation and invasion of T‐ALL cells by inhibiting the phosphatidylinositol 3‐kinase/AKT signaling pathway. The fat mass‐ and obesity‐associated protein (FTO), an m6A demethylase, is responsible for directly binding to m6A sites in 3′ untranslated region of *IRF8* messenger RNA (mRNA) and inducing mRNA degradation via m6A modification. Targeting the FTO‐IRF8 axis is used as a proof of concept therapy; inhibition of FTO's demethylase activity drastically alleviates the proliferation of leukemic cells and prolongs the survival of T‐ALL mice by restoring IRF8 expression. This study elucidates the pathogenesis of T‐ALL from the perspective of epitranscriptomics and provides new insight into the genetic mechanisms and targeted therapy of T‐ALL.

## Graphical abstract

Suppressed interferon regulatory factor 8 (IRF8) contributes to cell proliferation and leukemogenesis of T‐cell acute lymphoblastic leukemia (T‐ALL) via transcriptional regulation of PIK3R5 and activation of phosphatidylinositol 3‐kinase (PI3K)/AKT pathway. High expression of fat mass‐ and obesity‐associated protein (FTO) in T‐ALL inhibits IRF8 expression by binding to m6A sites in 3’ untranslated region (UTR) of *IRF8* messenger RNA (mRNA) and erasing m6A methylation modification, thereby promoting *IRF8* mRNA degradation.

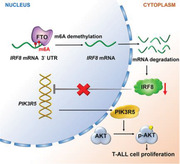

## 1 Introduction

T‐cell acute lymphoblastic leukemia (T‐ALL) is an invasive hematological disease that originates from clonal expansion of malignant lymphoid progenitor cells. With standard chemotherapies, only 40–50% of adult patients with T‐ALL survive for more than five years.^[^ ^1^, ^2^ ^]^ Worse yet, relapse occurs in ≈40% of adult patients with T‐ALL, leading to a long‐term overall survival rate of less than 7%.^[^ ^3^ ^]^ Novel studies have revealed the crucial role of genetic alteration in facilitating the initiation and progression of T‐ALL; for instance, mutations of the notch receptor 1 (*NOTCH1*) gene^[^ ^4^ ^]^ aberrantly activate the phosphatidylinositol 3‐kinase (PI3K)/AKT signaling pathway^[^ ^5^ ^]^ and epigenetic alternations.^[^ ^6^ ^]^ However, targeted therapies such as NOTCH1 and AKT inhibitors are presently unavailable for T‐ALL patients,^[^ ^7^, ^8^ ^]^ and common DNA demethylation agents such as decitabine exert limited therapeutic effect for T‐ALL.^[^ ^9^ ^]^ Thus, there is an imperative need to develop effective targeted therapies, urging for an in‐depth exploration into the genetic mechanisms underlying the pathobiology of T‐ALL.

Interferon regulatory factor 8 (IRF8) is a vital transcription factor for hematological lineage commitment.^[^ ^10^ ^]^ IRF8 is indispensable for the maintenance of myeloid cell development. Compromised IRF8 expression is recognized to be a pathogenic factor in myeloid leukemia.^[^ ^11^, ^12^ ^]^ Recently, the critical role of IRF8 in the development and function of lymphoid cells has been revealed. IRF8 deficiency blocks the transition of naïve CD8^+^ T cells into effector cells.^[^ ^13^ ^]^ Moreover, it serves as a negative regulator of T helper type 17 cell differentiation.^[^ ^14^ ^]^ Nevertheless, the roles and mechanisms of IRF8 in the pathogenesis of lymphoid malignancy remain unclear.

*N6*‐methyladenosine (m6A) modifications are the most prevalent epitranscriptomic modifications in eukaryotic messenger RNA (mRNA).^[^ ^15^, ^16^ ^]^ Recent studies have shown that m6A modification is a dynamic and reversible process that is involved in diverse biological processes.^[^ ^17^, ^18^ ^]^ m6A modification at the consensus motif of RRACH (R = G or A; H = A, C, or U) is modulated by the balanced coordination of m6A “writer”, “eraser”, and “reader” proteins.^[^ ^19^, ^20^ ^]^ “Writers” refer to the methyltransferase complex that is formed by methytransferase‐like 3 (METTL3) catalytic subunit as well as other auxiliary subunits such as methyltransferase‐like 14 (METTL14), Wilm's tumor 1‐accociating protein (WTAP), and RNA binding motif protein 15 (RBM15) and catalyzes m6A configuration.^[^ ^21^, ^22^, ^23^ ^]^ Conversely, “erasers” are demethylases that remove m6A, represented by the fat mass‐ and obesity‐associated protein (FTO) and alkB homolog 5 (ALKBH5).^[^ ^24^, ^25^ ^]^ Apart from altering the RNA structure, m6A could be recognized and bound by “readers,” such as YTH N6‐methyladenosine RNA binding proteins (YTHDFs) and insulin like growth factor 2 mRNA binding proteins (IGF2BPs), to control RNA metabolism, including translation, stability, splicing, folding and transport.^[^ ^26^, ^27^, ^28^ ^]^

Mounting evidence has revealed that the imbalance among the “writers”, “erasers”, and “readers” exerts a crucial role in leukemogenesis and progression. Increased abundance of m6A by METTL3 enhances cell growth and represses myeloid differentiation in acute myeloid leukemia (AML).^[^ ^29^, ^30^ ^]^ Meanwhile, FTO is involved in delayed differentiation and induces cellular apoptosis in AML.^[^ ^31^ ^]^ The essentiality and reversibility of m6A regulators make them promising therapeutic targets for small molecule inhibitors. For example, the highly selective and potent catalytic inhibitor of FTO (FB23‐2) exhibits significant repression of AML maintenance and prolonged the survival of AML recipients in vivo.^[^ ^32^ ^]^ However, there has been little research on the effect of m6A in T‐ALL.

In this study, we demonstrated that IRF8 was silenced in T‐ALL. Forced expression of IRF8 effectively inhibited the proliferation and invasion of T‐ALL cells, and knockout of Irf8 accelerated the development of Notch1‐induced T‐ALL in vivo via activating the PI3K/AKT signaling. Moreover, elevated FTO in T‐ALL reversibly reduced m6A modification of IRF8, thereby decreasing IRF8 expression by altering mRNA stability. Furthermore, inhibition of FTO by FB23‐2 effectively retrieved the expression of IRF8 and prolonged the survival of T‐ALL mice. Collectively, these results revealed the suppressive role of IRF8 in T‐ALL, and provide a new avenue of targeting epitranscriptomic modifying enzymes of *IRF8* mRNA as a promising alternative therapeutic strategy to overcome T‐ALL.

## 2 Results

### 2.1 IRF8 Expression Is Markedly Suppressed in T‐ALL Patients

**Figure 1:**
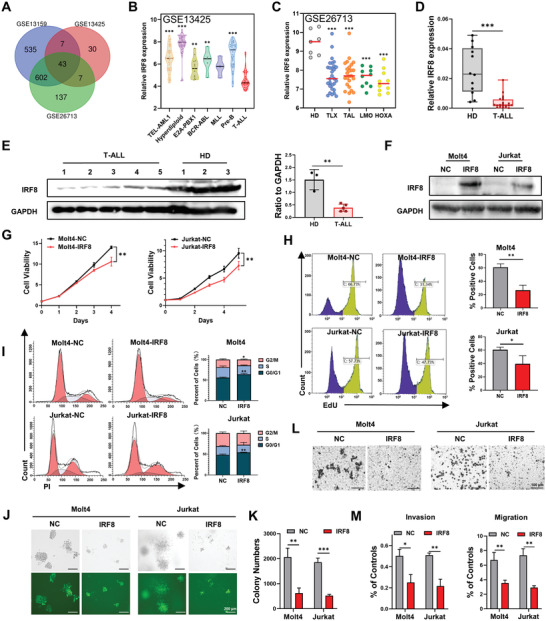
Suppressed IRF8 expression is responsible for the proliferation and invasion of T‐ALL. A) Venn diagram of DEGs from the three gene expression profile datasets (GSE13159, GSE26713, and GSE13425) downloaded from the GEO database. In GSE13159 and GSE26713, T‐ALL patients were compared with healthy donors, and in GSE13425, T‐ALL patients were compared with B‐ALL patients. B) IRF8 expression in patients with T‐ALL (*n* = 36) and other patients with ALL with various cytogenetic subtypes, versus patients with T‐ALL (GSE13425). C) IRF8 expression in patients with T‐ALL with TLX (*n* = 29), TAL (*n* = 25), LMO (*n* = 10) and HOXA (*n* = 10) mutations, compared with healthy donors (HD, *n* = 7, GSE26713). D) IRF8 expression in the bone marrow (BM) of newly diagnosed patients with T‐ALL (*n* = 23) and healthy donors (HD, *n* = 12) by qRT‐PCR. E) IRF8 expression in the BM of newly diagnosed patients with T‐ALL (*n* = 5) and healthy donors (HD, *n* = 3) by western blotting. F) Western blotting analysis of IRF8 levels in IRF8‐overexpressed Molt4 cells, Jurkat cells, and the corresponding negative control (NC) by lentiviral transfection. G) The proliferation analysis of IRF8‐overexpressed Molt4 cells, Jurkat cells, and NCs by CCK8 assays. H) DNA replication rates of IRF8‐overexpressed Molt4 cells, Jurkat cells, and their NC cells by EdU assays. I) Cell cycle analysis of IRF8‐overexpressed Molt4 cells, Jurkat cells, and NCs. J,K) Representative photographs (J) and statistical analysis (K) of the colony‐forming capacities of IRF8‐overexpressed Molt4 and Jurkat cells as well as NC cells. L) Representative photographs of crystal violet‐stained Molt4 and Jurkat cells overexpressing IRF8 and NCs on the underside of the membrane. M) The invasion and migration capacities of IRF8‐overexpressed Molt4 cells and NCs. (B) and (C): one‐way analysis of variance (ANOVA) with Dunnett's post hoc comparison; (D), (E), (H), (I), (K), and (M): unpaired, two‐tailed Student's test; (G): two‐way ANOVA with Sidak's post hoc comparison. Mean with standard deviation (SD), *n* = 3, *p < 0.05, **p < 0.01, ***p < 0.001.

To screen out the key molecules involved in the pathogenesis and progression of T‐ALL, the differentially expressed genes (DEGs) were analyzed in 3 datasets from multi‐center studies. In GSE13159 and GSE26713, T‐ALL patient cohorts were compared with healthy donors, and in GSE13425, T‐ALL patient cohorts were compared with B‐cell acute lymphoblastic leukemia (B‐ALL) patient cohorts. The overlapping of DEGs was further investigated, and 43 DEGs were identified, among which IRF8 was aberrantly suppressed in T‐ALL patients (**Figure** **1**A). Analysis of IRF8 expression in GSE13425 showed that IRF8 expression levels were markedly lower in T‐ALL than in B‐cell acute lymphoblastic leukemia (B‐ALL) with cytogenetic subtypes such as TEL‐AML1, hyperdiploid, and BCR‐ABL (Figure 1B). Moreover, in GSE26713, the expression level of IRF8 was relatively lower in T‐ALL patient cohorts with homeobox A (HOXA)‐activating rearrangement, which is considered an adverse prognostic factor, than in patients with T‐ALL and those with other genetic abnormalities (Figure 1C). Additionally, the mRNA and protein levels of IRF8 were significantly reduced in newly diagnosed T‐ALL patient cohorts enrolled in our hospital compared with healthy participants (*p* < 0.001, Figure 1D,E). A low level of IRF8 was also associated with shorter relapse‐free survival (RFS) (*p* < 0.05), elevated white blood cell (WBC) counts (*p* < 0.05), decreased platelet (PLT) counts (*p* < 0.01) and increased bone marrow (BM) blasts (*p* < 0.01) in T‐ALL patients (Figure S1A–H, Supporting Information).

### 2.2 IRF8 Inhibits the Proliferation and Invasion of T‐ALL Cells

To determine whether suppression of IRF8 is crucial to the survival of T‐ALL cells, we overexpressed IRF8 in T‐ALL cell lines, Molt4 and Jurkat, by lentiviral transfection. Upregulation of IRF8 was confirmed using western blotting analysis (Figure 1F). Cell counting kit 8 (CCK8) assay showed that upregulation of IRF8 significantly inhibited T‐ALL cell growth (Figure 1G). 5'‐Ethynyl‐2'‐deoxyuridine (EdU) assay revealed that, compared with the negative control (NC) group, the EdU positive rate of the IRF8‐overexpressed group was significantly decreased in Molt4 and Jurkat cells, indicating that IRF8 slowed down the DNA replication rate in T‐ALL cells (Figure 1H). Cell cycle analysis demonstrated that IRF8 overexpression induced a distinct G0/G1 phase cell cycle arrest and a concomitant decrease in the proportion of G2/M phase and S phase cells in Molt4 and Jurkat cells (Figure 1I). Besides, overexpression of IRF8 significantly impaired the colony formation ability. These results further consolidated the inhibition of cell proliferation by IRF8 (Figure 1J,K).

In addition, invasion assays showed that IRF8 suppressed the invasiveness of T‐ALL cells, demonstrated by a decreased penetration through the Matrigel‐coated membrane (Figure 1L,M). Moreover, the migration ability of T‐ALL cells was impaired by overexpression of IRF8 (Figure 1L,M).

### 2.3 Knockout of *Irf8* Promotes Notch1‐Induced T‐ALL Progression In Vivo

**Figure 2:**
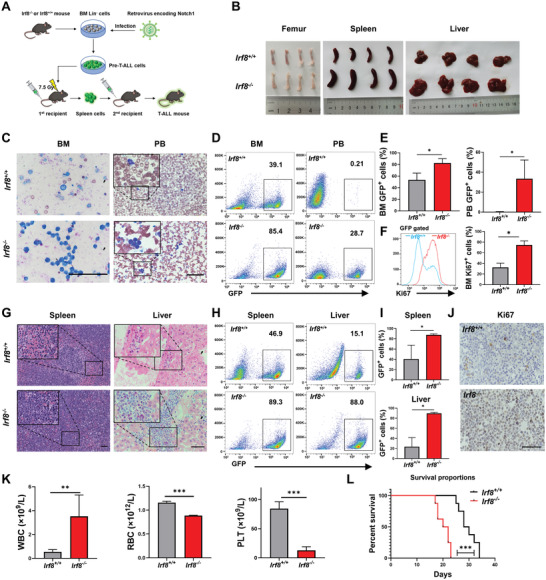
Knockout of *Irf8* accelerates the progression of Notch1‐induced T‐ALL. A) Schematic diagram of the procedures to establish the T‐ALL mouse model, a total of 4 original biological donor samples were used in each group. B) Images of femurs, spleens, and livers of mice transplanted with *Irf8^−/−^* and *Irf8^+/+^* spleen cells at two weeks post‐transplantation. C) Cell morphology of the bone marrow (BM) and peripheral blood (PB), detected by Wright‐Giemsa staining. D) Frequency of GFP^+^ cells in the BM and PB, detected by flow cytometry at two weeks post‐transplantation. E) Statistical analysis of GFP^+^ cells in the BM and PB (*n* = 4). F) Ki67 expression of GFP^+^‐gated bone marrow cells in the *Irf8^−/−^* and *Irf8^+/+^* groups at two weeks post‐transplantation (*n* = 4). G) Pathological sections of spleen and liver in the *Irf8^−/−^* and *Irf8^+/+^* groups. H) Flow cytometry determined the frequency of GFP^+^ cells in the spleen and liver at two weeks post‐transplantation. I) Statistical analysis of GFP^+^ T‐ALL cells in the spleen and liver (*n* = 4). J) Representative immunohistochemistry photograph of Ki67 expression in the spleen. K) White blood cell count (WBC), red blood cell count (RBC), and platelet (PLT) count in the PB at two weeks post‐transplantation. L) Survival of mice transplanted with *Irf8^−/−^* or *Irf8^+/+^* T‐ALL cells derived from the spleen (*n* = 8). Scar bar: 50 µm. (E), (F), (I), and (K): unpaired, two‐tailed Student's test; (L): Kaplan–Meier survival analyses with log‐rank test. Mean with SD, *n* = 3, *p < 0.05, **p < 0.01, ***p < 0.001.

Next, we sought to determine the effects of *Irf8* knockout on the development of T‐ALL in vivo. Bone marrow Lin^−^ cells from *Irf8^+/+^* or *Irf8^−/‐^* mice were sorted and infected with retrovirus expressing the intracellular domain of NOTCH1 (NICD) and green fluorescence protein (MSCV‐NICD‐IRES‐GFP). The transfected BM lineage‐negative (Lin^‐^) cells were then collected and transplanted intravenously into lethally irradiated C57BL/6 mice. Then, spleen cells with *Irf8^+/+^* or *Irf8^−/−^* were collected and transplanted into C57BL/6 mice (**Figure** **2**A). The difference in leukemic infiltration between *Irf8^+/+^* and *Irf8^−/−^* groups was the most obvious at Day 14 post‐transplantation (Figure S2A, Supporting Information). The results showed a highly aggressive development of T‐ALL in mice in the *Irf8* ^−/−^ group, with notably elevated penetrance and a shorter latent period than that in the *Irf8^+/+^* group. As shown in Figure 2B, the *Irf8^−/−^* group exhibited distinct hepatosplenomegaly and pale appearance of femurs, indicating leukocytosis, erythrocytopenia and infiltration in multi‐organs, while the *Irf8^+/+^* group showed leukemogenesis to a less extent. Fluorescence activating cell sorter (FACS) analysis and hematozlin and eozine stain (H&E) staining further showed higher frequencies of GFP leukemic lymphoblastic cells in the BM and blood of the *Irf8^−/−^* group than those in the *Irf8^+/+^* group (Figure 2C–E). Moreover, increased Ki67 levels in the BM GFP^+^ cells were found in the *Irf8^−/−^* group, suggesting that knockout of *Irf8* promoted the proliferation of leukemic cells (Figure 2F). Leukemic infiltrations in the spleen and liver were more obvious in the *Irf8^−/−^* group than in the *Irf8^+/+^* group, with increased GFP^+^ cells in the homogenate (Figure 2G–I). Immunohistochemical studies revealed an increase of Ki67^+^ cells in the spleen of T‐ALL mice in the *Irf8^−/−^* group (Figure 2J). Blood routine examination showed increased white blood cells (WBCs) in the *Irf8^−/−^* group, while erythroid cells and platelets were reduced (Figure 2K). As a result, T‐ALL mice in the *Irf8^−/−^* group had shortened median survival time than those in the *Irf8^+/+^* group (29 days versus 21 days, *p* < 0.001, Figure 2L). Taken together, these results revealed that loss of *Irf8* could act in collaboration with the driver oncogene to expedite the development of T‐ALL in vivo.

### 2.4 IRF8 Inhibits the Activation of the PI3K/AKT Pathway by Transcriptional Regulation of *PIK3R5*

**Figure 3:**
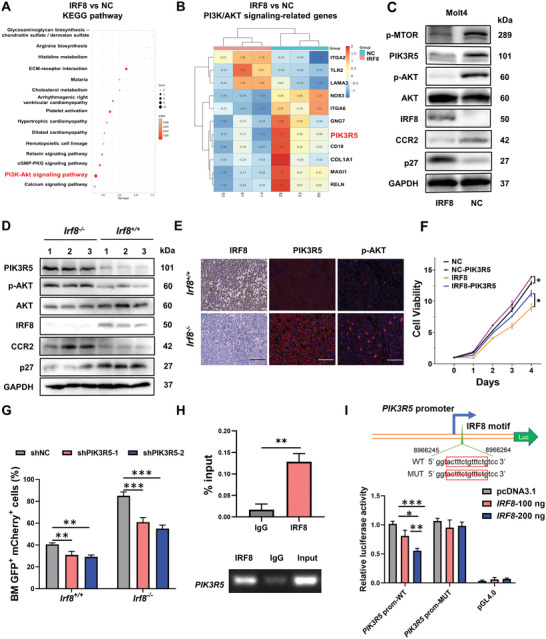
IRF8 suppresses PI3K/AKT pathway activation by inhibiting *PIK3R5* transcription. A) KEGG analysis showed enrichment of PI3K/AKT pathway in IRF8‐overexpressed Molt4 cells, compared with the negative control (NC) Molt4 cells, as detected by RNA‐seq. B) Heatmap shows DEGs related to PI3K/AKT signaling in IRF8‐overexpressed Molt4 cells (E1, F1, G1) and negative control (NC) Molt4 cells (E2, F2, G2). PIK3R5 was identified as a DEG (*p* < 0.05). C) Immunoblot analysis confirmed the altered expression of PI3K/AKT signaling and downstream molecules in IRF8‐overexpressed Molt4 cells compared with NC. D) Immunoblot analysis of PI3K/AKT signaling and downstream molecules in the BM cells from *Irf8^−/−^* and *Irf8^+/+^* T‐ALL mice (GFP^+^ cells over 80%). E) Representative immunofluorescence photographs of IRF8, PIK3R5, and p‐AKT expression in the spleen of *Irf8^−/−^* and *Irf8^+/+^* T‐ALL mice. F) Effects of PIK3R5 overexpression on the viability in Molt4‐NC and Molt4‐IRF8 cells. G) Effects of PIK3R5 knockdown on BM GFP+ mCherry+ cells in *Irf8* ^+/+^ and *Irf8* ^−/−^ T‐ALL mice at two weeks post‐transplantation. H) ChIP‐qPCR analysis of *PIK3R5* in Molt4 cells and the corresponding electropherogram. I) Schematic diagram of DNA fragments of *PIK3R5*‐promoter containing the wild‐type IRF8 motifs or corresponding deletion mutant of the predicted binding sequence, which was inserted in front of the luciferase reporter sequence; The relative luciferase activity of the wild‐type (*PIK3R5*‐prom‐WT) and the mutant (*PIK3R5*‐prom‐MUT) *PIK3R5* promoter reporter vectors in Molt4 cells with or without induced expression of *IRF8*‐pcDNA3.1 (100 or 200 ng). (F), (G), and (I): two‐way ANOVA with Tukey's post hoc comparison. (H): unpaired, two‐tailed Student's test. Mean with SD, *n* = 3, *p < 0.05, **p < 0.01, ***p < 0.001.

To unravel the molecular mechanism of how IRF8 affects the rapid proliferation of T‐ALL cells, IRF8‐overexpressed Molt4 cells and control cells were collected for RNA‐seq analysis. Kyoto Encyclopedia of Genes and Genomes (KEGG) analysis of DEGs revealed significant enrichment of the PI3K/AKT signaling pathway (*p* = 0.0019, **Figure** **3**A). Among the 11 DEGs identified as PI3K/AKT signaling‐associated genes, the phosphoinositide 3‐kinase regulatory subunit 5 (*PIK3R5*, also known as *P101‐PI3K*, Figure 3B) was further studied. *PIK3R5* encodes p101, a regulatory subunit of phosphoinositide 3‐kinase *γ* (PI3K*γ*), which is required for the catalytic activity of PI3Ks to phosphorylate AKT.^[^ ^33^ ^]^ Herein, the downregulation of PIK3R5 in IRF8‐overexpressed Molt4 cells was confirmed by immunoblot, along with the reduction of phosphorylated AKT (p‐AKT) levels and downstream phosphorylated mechanistic target of rapamycin kinase (p‐MTOR) levels. Additionally, the protein level of C‐C motif chemokine receptor 2 (CCR2) was also decreased, which explained the impaired invasiveness by IRF8 (Figure 3C). In the T‐ALL mouse models, T‐ALL cells from the *Irf8^−/−^* group showed increased expression of PIK3R5, which was consistent with the enhanced phosphorylation of AKT (Figure 3D). Moreover, p27, a downstream effector of PI3K/AKT, which undergoes proteasomal degradation mediated by p‐AKT, controls cell cycle progression and suppresses oncogenesis,^[^ ^34^ ^]^ was increased in IRF8‐overexpressed Molt4 cells and reduced in the *Irf8^−/−^* group (Figure 3C,D). Immunofluorescence analysis further confirmed that *Irf8* knockout enhanced the expression of PIK3R5 and p‐AKT (Figure 3E). To confirm that *PIK3R5* is responsible for the suppressive role of IRF8 in T‐ALL, rescue experiments were conducted in vivo and in vitro. CCK8 assay revealed that the overexpression of PIK3R5 significantly reversed the inhibition of cell proliferation caused by IRF8 overexpression (*p* < 0.05, Figure 3F and Figure S2B, Supporting Information). Similarly, knockdown of PIK3R5 in *Irf8^−/‐^* T‐ALL mice significantly reduced the proportion of GFP^+^ mCherry^+^ cells in the BM, suggesting that the knockdown of *PIK3R5* is efficient in rescuing the phenotype of increased leukemogenesis caused by *Irf8* depletion (Figure 3G and Figure S2C, Supporting Information). These results suggest that IRF8 negatively regulated the PI3K/AKT pathway via PIK3R5 in T‐ALL.

IRF8 functions as a transcriptional factor; hence, whether IRF8 directly targets PIK3R5 was verified using the Jaspar website. A potential binding site of IRF8 was identified in the promoter region of *PIK3R5* (Figure S2D, Supporting Information). Chromatin‐immunoprecipitation (ChIP) revealed significant enrichment of the *PIK3R5* promoter sequence in IRF8 immunoprecipitate as compared with IgG (0.13% versus 0.02% of input, Figure 3H), indicating that IRF8 could directly recognize the binding motif in the *PIK3R5* promoter sequence. Furthermore, a fragment of the *PIK3R5* gene 5′‐flanking region containing the potential binding motif (*PIK3R5*‐prom‐WT) or the corresponding deletion mutant fragment (*PIK3R5*‐prom‐MUT) were cloned in front of the firefly luciferase gene in the reporter plasmid. The reporter plasmids were co‐transfected with the *IRF8*‐expressing plasmid to perform luciferase assay. As shown in Figure 3I, IRF8 overexpression caused a reduction of luciferase activity in the reporter plasmid carrying the wild‐type *PIK3R5* promoter fragment in an IRF8 dose‐dependent manner, while the mutation of the IRF8‐binding site in the *PIK3R5* promoter remarkably abrogated this inhibition. These data supported the hypothesis that IRF8 directly regulated the transcription of *PIK3R5*.

### 2.5 IRF8 Is Silenced by the m6A‐Related Eraser FTO in T‐ALL

**Figure 4:**
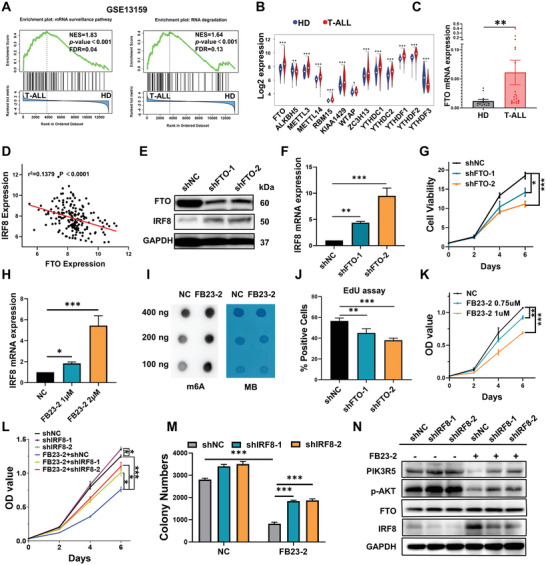
FTO regulates the expression and function of IRF8 in T‐ALL. A) GSEA of enriched gene sets in patients with T‐ALL from GSE13159 (*n* = 174). B) Log2 expression values of m6A regulators in the bone marrow of T‐ALL patients and healthy donors (HDs). Data was downloaded from GSE13159. C) FTO mRNA levels in the BM of newly diagnosed patients with T‐ALL (*n* = 23) and healthy donors (HD, *n* = 12), determined by qRT‐PCR, Mann–Whitney test. D) Correlation of expression levels between FTO and IRF8 across the 174 T‐ALL samples from GSE13159, Pearson correlation analysis. E) FTO and IRF8 levels in Molt4 cells transfected with FTO shRNA lentivirus (shFTO‐1 and shFTO‐2) or negative controls (shNC) by Western blot (*n* = 3). F) qRT‐PCR analysis of IRF8 mRNA levels in Molt4 cells transfected with shFTO‐1, shFTO‐2, or shNC lentivirus. G) Proliferation of Molt4 cells transfected with shFTO‐1, shFTO‐2, or shNC lentivirus. H) EdU positive rates of Molt4 cells transfected with shFTO‐1, shFTO‐2, or shNC lentivirus (*n* = 3). I) Dot blot analysis of m6A levels in Molt4 cells treated with 2 µm FB23‐2 or DMSO (NC) for 48 h. J) IRF8 mRNA levels of Molt4 cells treated with different concentrations of FB23‐2 or DMSO (NC) for 48 h. K) Proliferation of Molt4 cells treated with various concentrations of FB23‐2 or DMSO (NC). L) Viability of Molt4 cells transfected with shIRF8‐1, shIRF8‐2, or shNC lentivirus after FB23‐2 treatment. FB23‐2 treatment and shIRF8‐1, P‐interaction<0.01; FB23‐2 treatment and shIRF8‐2, P‐interaction < 0.001. M) The colony‐forming capacity of Molt4 cells with transfection of shIRF8‐1, shIRF8‐2, or shNC lentivirus treated with 1 µm FB23‐2. FB23‐2 treatment and shIRF8‐1, P‐interaction < 0.001; FB23‐2 treatment and shIRF8‐2, P‐interaction < 0.01. N) Immunoblot analysis of IRF8, PIK3R5 and p‐AKT levels in Molt4 cells with transfection of shIRF8‐1, shIRF8‐2 or shNC lentivirus incubated with 2 µm FB23‐2 for 48 h. (B): unpaired, two‐tailed Student's test; (F), (H), and (J): one‐way ANOVA with Dunnett's post hoc comparison; (G), (K), (L), and (M): two‐way ANOVA with Tukey's post hoc comparison. Mean with SD, *n* = 3, *p < 0.05, **p < 0.01, ***p < 0.001.

To further investigate why IRF8 is suppressed in T‐ALL, gene set enrichment analysis (GSEA) was conducted on the T‐ALL dataset GSE13159 (patients with T‐ALL versus healthy donors). Notably, multiple gene sets associated with the biological process of RNA were significantly enriched in T‐ALL patients, including the gene set of mRNA surveillance pathway and RNA degradation (**Figure** **4**A). m6A modification is recognized as the most prevalent internal modification in eukaryotic mRNA^[^ ^20^, ^21^ ^]^ that possesses functional importance in RNA fate and metabolisms,^[^ ^27^, ^30^, ^35^, ^36^ ^]^ therefore, the focus of this study was on m6A modification and regulators. Genetic expression analysis of GSE13159 revealed remarkable alteration of various m6A regulators in patients with T‐ALL, and the change in FTO levels was the most obvious (Figure 4B). Besides, qPCR assays confirmed that FTO expression in newly diagnosed T‐ALL patient cohorts was significantly upregulated than that in healthy donors (Figure 4C). A significant inverse correlation between FTO and IRF8 expression was indicated in two independent T‐ALL cohorts (*p* < 0.001 and *p* < 0.05, respectively, Figure 4D and Figure S2B, Supporting Information) by Pearson correlation analysis.

To verify whether FTO‐mediated m6A modification regulates IRF8 expression, FTO was knocked down by small hairpin RNA (shRNA) lentivirus. Western blot and qRT‐PCR showed that the knockdown of FTO resulted in an increased expression level of IRF8 (Figure 4E,F). Consequently, the suppression of cell proliferation and inhibition of DNA replication in Molt4 cells were observed (Figure 4G,H). Additionally, FB23‐2, a novel small molecular inhibitor of FTO, was used to inhibit FTO activity. FB23‐2 binds to FTO directly and suppresses the enzymatic activity of m6A demethylase selectively, which is considered to have great potential and advantages in the field of epitranscriptomic RNA methylation‐targeted therapy.^[^ ^32^ ^]^ Dot blot assay showed that FB23‐2 treatment enhanced the mRNA m6A level in Molt4 cells (Figure 4I). Correspondingly, the expression level of IRF8 was elevated, and cell proliferation was inhibited by FB23‐2 in a dose‐dependent manner (Figure 4J,K). Moreover, the inhibition of cell proliferation by FB23‐2 could be effectively rescued by the knockdown of IRF8 (Figure 4L). Consistently, the knockdown of IRF8 could reinstate the colony formation ability of Molt4 cells inhibited by FB23‐2 treatment (Figure 4M). A significant interaction between FB23‐2 treatment and IRF8 knockdown was also demonstrated in cell viability (FB23‐2 treatment and shIRF8‐1, P‐interaction < 0.01; FB23‐2 treatment and shIRF8‐2, P‐interaction < 0.001, Figure 4L) and in colony formation activity (FB23‐2 treatment and shIRF8‐1, P‐interaction < 0.001; FB23‐2 treatment and shIRF8‐2, P‐interaction < 0.01, Figure 4M) by factorial analysis. In addition, western blotting analysis showed that the FB23‐2 treatment restored IRF8 expression while inhibiting the expression of PIK3R5 and activation of AKT (Figure 4N). These findings provide strong evidence that the expression of IRF8 were negatively regulated by FTO‐mediated m6A modification.

### 2.6 FTO Negatively Regulates IRF8 by an m6A‐Dependent Mechanism

**Figure 5:**
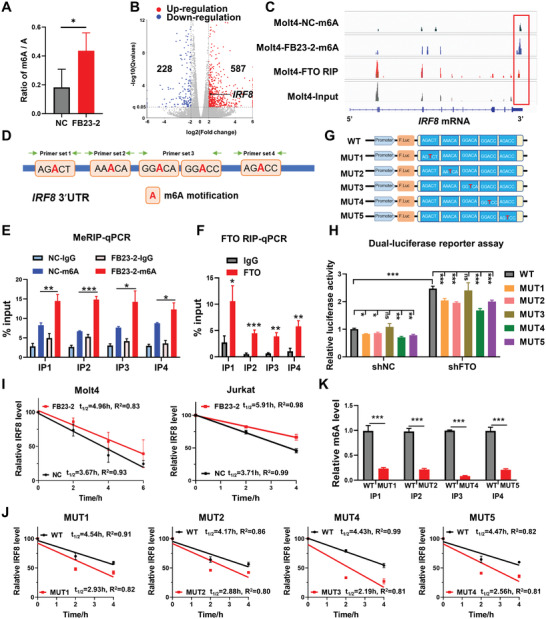
FTO suppresses IRF8 by an m6A dose‐dependent mechanism. A) Global m6A abundance of mRNA in Molt4 cells incubated with 2 µm FB23‐2 or DMSO (NC) for 48 h, described as the ratio of m6A/A by LC‐MS/MS. B) Volcano plot of DEGs; IRF8 was labeled out in black dot. Blue dots (*n* = 228): downregulated genes; Red dots (*n* = 587): upregulated genes. C) Integrative Genomics Viewer (IGV) tracks depicted the m6A modification of *IRF8* gene in Molt4 cells treated with DMSO vehicle or FB23‐2, as well as the FTO RIP‐seq signal and the corresponding input signal in the *IRF8* locus. D) Schematic diagram of potential m6A binding sites (Site 1–5) in IRF8 3′ UTR. Primer sets 1–4 are represented with IP1‐4 in the following content. Primer set 3 (IP3) includes 2 sites: Site 3 and Site 4. E) MeRIP‐qPCR analysis of m6A levels of *IRF8* mRNA in Molt4 cells treated with or without FB23‐2 for 48 h. Primer sets 1–4 are represented with IP1‐4. F) FTO RIP‐qPCR analysis showed the binding of FTO to *IRF8* mRNA transcripts in Molt4 cells. Primer sets 1–4 are represented with IP1‐4. G) Schematic diagram of wild‐type 3′ UTR of *IRF8* mRNA (WT) cloned into pMIR‐REPORT luciferase vector as well as five mutant sequences where the m6A recognition sites were mutated from the A nucleotide to T nucleotide (MUT1 to MUT5). H) Relative luciferase activity of the wild‐type 3′ UTR of *IRF8* mRNA (WT) and the mutant 3′ UTR of *IRF8* mRNA (MUT1 to MUT5) reporter vectors transfected HEK293T cells with or without FTO knockdown. I) *IRF8* mRNA degradation assay of Molt4 and Jurkat cells pretreated with 5 µm FB23‐2 or DMSO (NC) for 24 h and incubated with 5 µg mL^−1^ Act‐D at indicated time before harvest, and normalized to mRNA levels at 0 h. J) RNA stability assays in HEK293T cells overexpressing wild‐type 3′ UTR of *IRF8* mRNA (WT) or mutant 3′ UTR of *IRF8* mRNA (MUT 1, MUT2, MUT4, and MUT5), incubated with 5 µg mL^−1^ Act‐D before harvest. K) MeRIP‐qPCR analysis of m6A levels in mRNA transcripts of wild‐type *IRF8* 3′ UTR (WT) or mutant *IRF8* 3′ UTR (MUT 1, MUT2, MUT4, and MUT5) in HEK293T cells, normalized to m6A levels of the WT group. (A), (F) and (K): unpaired, two‐tailed Student's test; (E): one‐way ANOVA with Dunnett's post hoc comparison; (H): two‐way ANOVA with Tukey's post hoc comparison. Mean with SD, *n* = 3, *p < 0.05, **p < 0.01, ***p < 0.001; ns, not significant.

To further validate that IRF8 was the downstream substrate of FTO, we performed RNA‐seq, methylated RNA immunoprecipitation (MeRIP)‐seq of Molt4 cells treated with or without the FTO inhibitor FB23‐2, as well as FTO RIP‐seq assays in Molt4 cells. Liquid chromatography‐tandem mass spectrometry (LC‐MS/MS) assays showed that the overall m6A abundance was enhanced after FB23‐2 treatment (**Figure** **5**A). Principal component analysis (PCA) revealed that Molt4 cells treated with FB23‐2 exhibited distinct gene expression characteristics compared with the controls (Figure S3A, Supporting Information). After FB23‐2 treatment, 587 genes (72.02%) were significantly upregulated, among which *IRF8* expression was elevated by two to three fold (Figure 5B and Figure S3B, Supporting Information). MeRIP‐seq analysis of Molt4 cells treated with or without FB23‐2 revealed that inactivation of FTO led to a significant increase in the m6A level of *IRF8* 3′ untranslated region (UTR). Moreover, the FTO RIP‐seq analysis revealed that FTO binding peaks were enriched in *IRF8* mRNA transcripts, and the tracks in the 3′ UTR of *IRF8* transcripts overlapped with the m6A peaks (Figure 5C). Motif analysis showed that the RRACH (R = G or A; H = A, C, or U) motif was highly enriched within the m6A sites in Molt4 cells (Figure S3C, Supporting Information). In addition, MeRIP‐seq data of FTO‐overexpressed leukemic cells downloaded from GSE76414 further consolidated that overexpression of FTO leads to a significant suppression in the level of m6A modification of *IRF8* 3′ UTR (Figure S3D, Supporting Information).

To confirm that *IRF8* is a critical target of m6A mRNA modification mediated by FTO, primers were designed to amplify sequences containing the potential binding sites predicted by motif analysis in *IRF8* 3′ UTR (Figure 5D). MeRIP‐qPCR assays were performed to select the m6A sites that were significantly enriched after FB23‐2 treatment (Figure 5E), followed by FTO RIP‐qPCR to select potential m6A sites that could be directly bound by FTO (Figure 5F). Subsequently, five potential m6A sites were obtained (Site 1–5). Afterward, the wild‐type *IRF8* 3′ UTR (WT) reporter vector and five mutant *IRF8* 3′ UTR reporter vectors were constructed by replacing each of the candidate m6A sites from A to T (MUT1 to MUT5, Figure 5G). The luciferase activities in WT reporter vector transfected HEK293T‐shFTO cells were significantly higher than HEK293T‐shNC cells (*p* < 0.001). Transfection of MUT1, MUT2, MUT4, and MUT5 reporter vectors remarkably restored the luciferase activities (Figure 5H). This result indicated that IRF8 is a critical target of FTO, which negatively regulates the *IRF8* mRNA expression via these m6A sites (Site 1, Site 2, Site 4, and Site 5).

### 2.7 FTO Regulates IRF8 Expression by Affecting RNA Stability

Whether FTO regulated *IRF8* expression by altering RNA stability was further investigated. Molt4 and Jurkat cells were pretreated with FB23‐2, followed by the transcription inhibitor actinomycin D (Act‐D). A remarkable increase in the half‐life of *IRF8* transcript after inhibition of FTO by FB23‐2 was observed, indicating that the inhibition of the FTO demethylation activity restored the *IRF8* mRNA expression by promoting RNA stability and reducing RNA degradation (Figure 5I).

Furthermore, to determine whether mutations in m6A sites of *IRF8* transcripts affect transcript stability, RNA stability assays were carried out in HEK293T cells overexpressing the wild‐type 3′ UTR of *IRF8* mRNA (WT) or mutant 3′ UTR of *IRF8* mRNA (MUT 1, MUT2, MUT4, and MUT5) with the primers that specifically amplify the corresponding fragments. The results revealed that mutations in m6A sites remarkably shortened the half‐life of *IRF8* mRNA transcripts (Figure 5J). In addition, to ensure that altered m6A modifications caused the effects observed above, MeRIP‐qPCR was performed to determine the m6A levels on 3′ UTR of *IRF8* mRNA in wild‐type or mutant *IRF8* 3′ UTR‐overexpressed HEK293T cells. It was confirmed that mutations in these m6A sites significantly reduced the m6A levels on 3′ UTR of *IRF8* mRNA (*p* < 0.001, Figure 5K). Collectively, these results indicate that FTO negatively regulated the mRNA stability of *IRF8* by alteration of m6A modification.

### 2.8 FTO Inhibitor Restores *IRF8* Expression, Inhibits PI3K/AKT Pathway, and Exhibits Anti‐Leukemic Effects In Vivo

**Figure 6:**
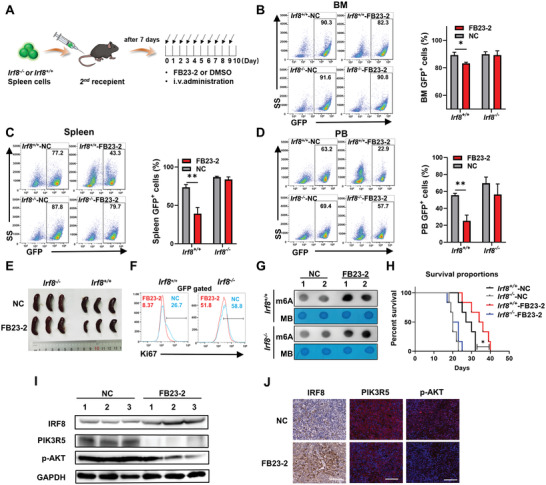
Upregulation of IRF8 by pharmacological inhibition of FTO alleviates the development of T‐ALL in vivo. A) Schematic diagram of establishing *Irf8^−/−^* and *Irf8^+/+^* T‐ALL mouse models and the schedule of FB23‐2 administration (FB23‐2: 2 mg kg^−1^). B) Proportion of GFP^+^ cells in the BM of *Irf8^−/−^* and *Irf8^+/+^* T‐ALL mice (*n* = 3) treated with FB23‐2 or DMSO (NC), as detected by flow cytometry. C) Proportion of GFP^+^ cells in the spleen (*n* = 3). D) The proportion of GFP^+^ cells in the peripheral blood (*n* = 3). E) Photograph of spleens of *Irf8^−/−^* and *Irf8^+/+^* T‐ALL mice treated with FB23‐2 or DMSO (NC). F) Ki67 expression levels in GFP^+^‐gated bone marrow cells, determined with flow cytometry. G) Dot blot analysis of m6A levels in spleen cells. H) Survival of mice in *Irf8^−/−^* and *Irf8^+/+^* groups treated with FB23‐2 or DMSO (NC) (*n* = 6). I) Immunoblot analysis of IRF8, PIK3R5, and p‐AKT levels in the BM of the *Irf8^+/+^* T‐ALL mice with the treatment of FB23‐2 or DMSO (NC). J) Representative immunofluorescence photographs of IRF8, PIK3R5, and p‐AKT expression in the spleen of the *Irf8^+/+^* T‐ALL mice treated with FB23‐2 or DMSO (NC). Scar bar: 50 µm. (B), (C) and (D): unpaired, two‐tailed Student's test; (H): Kaplan–Meier survival analyses with log‐rank test. Mean with SD, *n* = 3, *p < 0.05, **p < 0.01.

To verify the regulation of *IRF8* by FTO in vivo and explore the therapeutic potential of FTO inhibitor in T‐ALL, T‐ALL mouse models in *Irf8* ^+/+^ and *Irf8* ^−/‐^ groups were treated with FB23‐2. As shown in **Figure** **6**A, T‐ALL mice were intravenously injected with FB23‐2 daily for 10 days at a dosage of 2 mg kg^−1^. At the end of the treatment, T‐ALL mice were sacrificed to examine the leukemic burden at Day 19 post‐transplantation. At this time, the proportion of GFP^+^ T‐ALL cells in the bone marrow of both *Irf8* ^−/−^ and *Irf8* ^+/+^ groups without FB23‐2 treatment had reached peaks (Figure S2A, Supporting Information). Flow cytometry showed that FB23‐2 treatment significantly reduced the proportion of GFP^+^ cells in the BM, blood and spleen of the *Irf8* ^+/+^ group, while no difference was found in the *Irf8* ^−/−^ group (Figure 6B–D). Splenomegaly was also alleviated by FB23‐2 in the *Irf8* ^+/+^ group (Figure 6E). Besides, the Ki67 levels of GFP^+^ cells in bone marrow were markedly inhibited after FB23‐2 treatment in the *Irf8* ^+/+^ group (Figure 6F). Dot blot assay revealed significant enhancement in the m6A abundance in both groups (Figure 6G). The survival time of T‐ALL mice was prolonged in the *Irf8* ^+/+^ group after FB23‐2 treatment, while the therapeutic effect was not apparent in mice in the *Irf8* ^−/−^ group (Figure 6H). The above results suggested that FB23‐2 could efficiently alleviate the leukemic burden and prolong the survival of *Irf8* ^+/+^ T‐ALL mice, probably depending on the IRF8 expression. Immunoblot and immunofluorescence analysis revealed that FB23‐2 significantly increased the IRF8 expression of BM and spleen leukemic cells in the *Irf8* ^+/+^ group, as well as suppressed the expression of PIK3R5 and phosphorylation of AKT (Figure 6I,J). Based on the above results, we suggest that the pharmacological inhibition of m6A demethylase activity of FTO by FB23‐2 successfully restored the IRF8 expression in T‐ALL in vivo, thus inhibiting the process of leukemogenesis via inhibition of PI3K/AKT signaling.

## 3 Discussion

The current therapeutic effects and prognosis of T‐ALL remain poor, requiring the development of new therapeutic targets and treatment. In the present study, IRF8 played a key inhibitory role in the progression of T‐ALL. Abnormal silencing of IRF8 is involved in various tumors and hematological malignancies,^[^ ^11^, ^37^, ^38^ ^]^ highlighting its role as a tumor suppressor gene. Previous results mainly focus on the methylation and acetylation modifications of *IRF8*.^[^ ^39^, ^40^, ^41^ ^]^ However, the poor sensitivity of demethylating and deacetylating agents limits their clinical applications in treating T‐ALL. In recent years, the boom of epitranscriptomic studies has provided new methods to manipulate *IRF8* expression. m6A modification is acknowledged as the most prevalent epitranscriptomic modification in mammalian mRNA. Dysregulation of m6A modification shows a strong association with many malignancies,^[^ ^24^ ^]^ but the role of m6A in T‐ALL remains to be established. Here, we systematically analyzed the expression of m6A regulators in T‐ALL. A variety of regulators were found to be abnormally expressed in T‐ALL, of which FTO was highly upregulated.

FTO is the first identified m6A demethylase, which is responsible for eliminating the m6A modification from target mRNA.^[^ ^42^ ^]^ The current study showed that highly‐expressed FTO in T‐ALL negatively altered *IRF8* mRNA expression by affecting the *IRF8* mRNA stability, which highly relied on its m6A catalytic activity. Epidemiologic studies reveal a strong correlation between increased FTO expression and single nucleotide polymorphism (SNP) risk genotypes in various cancers, such as breast cancer^[^ ^43^ ^]^ and melanoma.^[^ ^44^ ^]^ Notably, a negative association between the FTO rs9939609 T allele and obesity was found in childhood ALL survivors.^[^ ^45^ ^]^ Therefore, upregulation of FTO is potentially caused by the aberrant distribution of SNPs in T‐ALL.

The role of FTO to reduce the mRNA stability in an m6A‐dependent manner was demonstrated recently. FTO promotes the leukemogenesis of AML by removing m6A in retinoic acid receptor alpha and ankyrin repeat and SOCS box containing 2 mRNA, inhibiting the mRNA stability, and finally decreasing mRNA expression.^[^ ^31^ ^]^ Another study suggested that FTO promotes the degradation of BCL2 interacting protein 3 (BNIP3) mRNA by binding to the m6A site in the 3′ UTR of *BNIP3* in breast cancer.^[^ ^46^ ^]^ Although previous studies suggest that m6A controls mRNA stability through YTHDF2‐mediated mRNA destabilization,^[^ ^47^ ^]^ emerging evidence indicates that novel m6A readers, such as IGF2BPs proteins^[^ ^48^ ^]^ and heterogeneous nuclear ribonucleoprotein A2/B1^[^ ^49^ ^]^ can bind to specific m6A sites to enhance mRNA stability. Our study brings new evidence that FTO negatively regulates gene expression via erasing m6A modification, revealing the complicated role of m6A modification in controlling RNA stability.

The development of potent FTO inhibitors shed light on the epitranscriptomic regulation of *IRF8* for the treatment of T‐ALL. FB23‐2 was recently designed as a highly‐potent selective FTO inhibitor, which directly binds to the FTO catalytic domain and efficiently represses the demethylase activity. FB23‐2 exerts remarkable inhibition of leukemic proliferation in AML cells and mouse models.^[^ ^32^ ^]^ Herein, we demonstrated that FB23‐2 effectively suppressed tumor progression in T‐ALL cells and Notch1‐induced T‐ALL mice by elevating m6A modification. Moreover, the therapeutic function of FB23‐2 was significantly reversed by the downregulation of IRF8, indicating that IRF8 is indispensable to achieving the therapeutic efficacy of FB23‐2 in T‐ALL. Notably, FB23‐2 treatment exhibited notable suppression of leukemic infiltration in the peripheral blood and in the extramedullary organs of Notch1‐induced T‐ALL mouse models but showed moderate inhibition in the bone marrow. Biological barriers inside the bone marrow microenvironment hinder drug delivery to the bone marrow.^[^ ^50^ ^]^ Thus, developing an elaborate bone marrow‐targeted drug delivery system is necessary to achieve a therapeutic concentration of FB23‐2 in the bone marrow.

To decipher the underlying mechanism by which IRF8 inhibits leukemogenesis in T‐ALL, the transcriptomic profiling of stable IRF8‐overexpressed Molt4 cells and control cells was analyzed. Subsequently, the inactivation of PI3K/AKT signaling was confirmed. ChIP and luciferase assays further confirmed that IRF8 is directly bound to *PIK3R5*, which encodes for a regulatory subunit of PI3K*γ*, to repress its transcription, thus inhibiting the phosphorylation of AKT.^[^ ^51^ ^]^ PIK3R5 contributes to the survival of T‐ALL cells by activating the PI3K*γ*/p‐AKT signaling.^[^ ^52^ ^]^ Moreover, its oncogenic role is indicated in T‐cell lymphoma.^[^ ^53^ ^]^ PI3K/AKT signaling is activated in more than 90% and 80% of T‐ALL cell lines and primary T‐ALL samples, respectively, and greatly contributes to T‐ALL pathogenesis.^[^ ^54^, ^55^ ^]^ Here, IRF8 functioned as a transcriptional factor to regulate the transcription of downstream molecules. The N‐terminal region of IRF8 protein is a well‐conserved N‐terminal DNA‐binding domain, which participates in the regulation of gene transcription by binding to the core IRF binding motif, GAAA. The C‐terminal region contains the IRF association domain, which is a less well‐conserved protein–protein interaction module to respond to the recruitment of Ets transcription factors or IRFs.^[^ ^56^ ^]^ FB23‐2 treatment inhibited the PI3K/AKT signaling pathway, which was consistent with the upregulated IRF8 level. Furthermore, the effect could be partially restored by inhibition of IRF8, suggesting that the regulation of PI3K/AKT signaling by FTO is, at least, partially mediated by IRF8.

## 4 Conclusion

Collectively, our study shows a novel gene regulation mechanism in T‐ALL that provides in‐depth insights into the molecular mechanism of leukemic pathogenesis. As the silencing of *IRF8* has also been implicated in many other malignancies, our findings possess a high potential to be extrapolated in pathological research and targeted therapy of T‐ALL.

## 5 Experimental Section

### Bioinformatics Analysis

Three gene expression profile datasets (GSE13159, GSE26713, and GSE13425) were downloaded from the GEO database. RStudio (version 1.1) was applied to analyze the data with the Affymetrix package and Limma package. Threshold criteria were set up as logFC ≥ 1.5 and adjust *p* < 0.01 were applied to achieve the fold‐change (FC) of gene expression for GSE13159 and GSE26713, and logFC ≥ 2 and adjust *p* < 0.01 for GSE13425. The Venn diagram analysis for DEGs was performed with the online tool Bioinformatics & Evolutionary Genomics (http://bioinformatics.psb.ugent.be/webtools/Venn/). GSEA was performed to determine the enriched gene sets in GSE13159 using the online tool: WEB‐based GEne SeT AnaLysis Toolkit (http://www.webgestalt.org/option.php).

### Primary Samples and Cell Lines of T‐ALL

BM samples were obtained from newly‐diagnosed T‐ALL patients (*n* = 23) from June 2014 to November 2021 at Qilu Hospital, Cheeloo College of Medicine, Shandong University, Jinan, China. Control samples were obtained from healthy donors (*n* = 12). All procedures with primary samples were approved by the Medical Ethics Committee of Qilu Hospital, Cheeloo College of Medicine, Shandong University (KYLL‐2017(KS)‐197). Informed consent was obtained following the Helsinki Declaration and national laws. The clinical characteristics of the patients are listed in Table S1, Supporting Information.

The human T‐ALL cell lines, Molt4 and Jurkat, were obtained from the Institute of Hematology & Blood Diseases Hospital, Chinese Academy of Medical Sciences & Peking Union Medical College, Tianjin, China. Cells were cultured under the condition of 10% fetal bovine serum (FBS, Gibco) and 1% penicillin‐streptomycin (Invitrogen) added RPMI 1640 medium and maintained at 37 °C with 5% CO_2_ atmosphere.

### Lentiviral Transfection

Lentiviral constructs expressing IRF8 and GFP (GV492‐IRF8‐GFP), along with the negative control lentivirus, were purchased from Genechem (Shanghai, China). GFP‐expressing lentiviral constructs of shIRF8 or mCherry‐expressing lentiviral constructs of shFTO and negative control lentivirus were purchased from GenePharma (Shanghai, China). mCherry‐expressing lentiviral constructs of shPIK3R5, PIK3R5, and negative control lentivirus were purchased from RiboBio (Guangzhou, China). The sequences are listed in detail in Table S2, Supporting Information. Cells were infected with lentivirus for 48 h and selected afterward using flow cytometry.

### Construction of Notch1‐Induced T‐ALL Mouse Model

*Irf8^+/‐^* C57BL/6 mice were obtained from the Jackson Laboratory and were bred and maintained under specific pathogen free (SPF) conditions. All animal experiments were carried out in accordance with the Animal Management Rules of the Ministry of Health of the People’s Republic of China. All experiments were authorized by the Laboratory Animal Ethical and Welfare Committee of Qilu Hospital, Cheeloo College of Medicine, Shandong University (Approval NO. DWLL‐2020‐016). *Irf8* ^−/−^ or *Irf8* ^+/+^ mice were bred in Gem Pharmatech (Nanjing, China). Retrovirus encoding NICD and GFP (MSCV‐NICD‐IRES‐GFP) were prepared by co‐transfecting the MSCV‐NICD‐IRES‐GFP plasmid to HEK293T cells along with two packaging plasmids pKat and VSVG, which were kindly provided by Professor Cheng Tao of the Institute of Hematology, Chinese Academy of Medical Science. The HEK293T cell culture supernatant containing retrovirus was collected and concentrated for further treatment. Bone marrow cells from *Irf8* ^−/−^ or *Irf8* ^+/+^ mice were dissected from femurs and tibias and sorted with Lin‐coated magnetic beads to obtain Lin^−^ cells. Retroviral infection was performed by adding the supernatant into Lin^−^ cells. Green fluorescence was observed 48 h later. Subsequently, 2 × 10^5^ GFP^+^ Lin^−^ cells were intravenously injected into lethally irradiated (7.5 Gy) 8‐12‐week recipient C57BL/6 mice (SiPeiFu Biotechnology, China) to establish the bone marrow transplant T‐ALL models. Furthermore, *Irf8* ^−/−^ or *Irf8* ^+/+^ leukemic cells were collected from the spleen of T‐ALL mice that exhibited over 80% of GFP^+^ cells in the spleen and were transplanted intravenously into C57BL/6 mice to investigate the function of *Irf8* in the occurrence and development of T‐ALL. The overall survival time and leukemic cell progression of mice were periodically monitored.

### ChIP‐qPCR

ChIP assay was applied using the SimpleChIP Enzymatic Chromatin IP Kit (CST, USA) based on the manufacturer's protocols. In short, chromatin fragments were extracted from Molt4 cells. Immunoprecipitation was performed using 5 µg of IRF8 antibody (CST, USA) or IgG antibody. Each ChIP eluate was amplified by qPCR for the 5′‐upstream region of the human *PIK3R5* gene (*PIK3R5*‐Promoter) using the primers shown in Table S2, Supporting Information. 2^−ΔCt^ of eluate relative to the input sample were calculated and analyzed.

### MeRIP‐Sequence

Total RNA from FB23‐2 (2 µm, AbMole, China) treated Molt4 cells and control cells was isolated and purified using TRIzol reagent (Invitrogen, Carlsbad, CA, USA) following the manufacturer's procedure. Poly (A) RNA was purified from 50 µg total RNA using Dynabeads Oligo (dT)25‐61005 (Thermo Fisher, CA, USA) by two rounds of purification. Poly (A) RNA fragmentation was performed using Magnesium RNA Fragmentation Module (NEB, cat.e6150, USA) to break down RNA randomly. m6A‐IP and input samples were prepared for generating libraries. Paired‐end sequencing (PE150) was performed on an Illumina Novaseq 6000 platform (LC‐Bio Technology CO., Ltd., China). Sequence reads were aligned to human genome version 38 with HISAT2. Peak calling was performed to identify differential m6A modified peaks by analyzing MeRIP‐seq bam files using exomePeak. Motif analysis of the m6A modified peaks was performed using HOMER.

### MeRIP‐qPCR

Total RNA was extracted from FB23‐2 treated or control Molt4 cells with TRIzol reagent. Immunoprecipitation of m6A‐containing mRNAs was carried out using Methylated RNA Immunoprecipitation Kit (BersinBio, China). qRT‐PCR was performed to quantify the input RNA, isolated m6A‐containing RNA, and IgG RNA using a 2 × SYBR Green Pro Taq HS Premix (AG, China) and run on Roche Light Cycler 480 II (Roche, Switzerland). *IRF8* primers were designed to amplify the region containing the m6A peak. IgG antibody was used to prepare the negative controls. 2^−ΔCt^ values were calculated to access the RNA expression of eluate relative to the input samples.

### RIP‐qPCR and RIP Sequence

RIP‐qPCR experiment was performed using Magna RIP Kit (Merck Millipore, USA) in Molt4 cells according to the instructions. Briefly, cell lysate was prepared as 5 × 10^7^ cells per sample. Protein A/G MagBeads were pre‐coated with 5 µg of the FTO antibody (ABclonal, China) for 30 min at room temperature, followed by incubation with cell lysate supernatant at 4 °C overnight. Specific binding RNAs were acquired from the immunoprecipitated RNA‐protein complex by using Proteinase K buffer to digest the proteins, and isolated by using TRIzol. qRT‐PCR were performed to quantify the RNA expression. FTO RIP samples and input samples were prepared for generating libraries. Paired‐end sequencing (PE150) was performed on an Illumina Novaseq 6000 platform (LC‐Bio Technology CO., Ltd., China). Data were analyzed as previously described.

### Dual‐Luciferase Reporter Assay

To clarify the effect of IRF8 on *PIK3R5* expression and identify the binding sites, DNA fragments of *PIK3R5*‐promoter containing the wild‐type IRF8 motifs (*PIK3R5*‐prom‐WT) and the mutant *PIK3R5*‐promoter with deletion mutation of the potential binding sequence (*PIK3R5*‐prom‐MUT) were synthesized by OBiO Technology (China). For dual‐luciferase reporter assay, 100 or 200 ng pcDNA3.1‐*IRF8* (or pcDNA3.1 empty vector), 200 ng pGL4.10‐*PIK3R5*‐prom‐WT (or pGL4.10‐*PIK3R5*‐prom‐MUT or pGL4.10), and 20 ng pRL‐TK (Renilla luciferase control plasmid) were co‐transfected into HEK293T cells in a 24‐well plate. 48 h later, the luciferase activities were measured by a Synergy H1 Hybrid Microplate Reader (BioTek, USA).

To determine how FTO regulates mRNA expression of *IRF8*, and identify the recognition sites, the 3′ UTR of *IRF8* mRNA was cloned into the pMIR‐REPORT Luciferase vector (WT). Five putative m6A recognition sites were identified previously and mutated from A into T to generate the corresponding mutant plasmids (MUT1‐MUT5). 100 ng of the above plasmids (or empty vector) were co‐transfected with 20 ng pRL‐TK into FTO‐knockdown HEK293T cells (HEK293T‐shFTO) and control cells (HEK293T‐shNC) in a 24‐well plate, relatively. 48 h later, the luciferase activities were measured as mentioned above.

### RNA Stability Assays

Molt4 and Jurkat cells were seeded in a 6‐well plate and pre‐treated with FB23‐2 (5 µm) or DMSO for 24 h. Then actinomycin D (AbMole, China) was added at a concentration of 5 µg mL^−1^ at the indicated time before harvest. Total RNA was isolated by TRIzol reagent. qRT‐PCR was performed to access the relative mRNA expression of *IRF8* (normalized to *18S rRNA*) with a Roche Light Cycler 480 II (Roche, Switzerland). A linear regression model was established to analyze the half‐time (*t*_1/2_) of RNA degradation by using GraphPad Prism 8.0 (GraphPad Software, USA).

### Statistical Analysis

Mean ± standard deviation (SD) values were calculated and analyzed using GraphPad Prism 8.0. Three independent experiments were performed unless otherwise specified. Differences in the mean values between the two indicated groups were analyzed using an unpaired two‐tailed Student's *t*‐test. One‐way analysis of variance (ANOVA) with Dunnett's post‐hoc comparison was utilized to evaluate the statistical significance between 3 or more groups. Two‐way ANOVA with Sidak's or Tukey's post hoc comparison was performed to compare the difference between 2 or more groups with different time points, or determine the interaction between two independent variables. Kaplan–Meier survival analyses were performed with the log‐rank test. *p* < 0.05 was considered statistically significant.

## Conflict of Interest

The authors declare no conflict of interest.

## Author Contributions

Y.Z. and M.J. contributed equally to this work. M.J., J.Y., and C.J. designed the research; Y.Z., M.J., X.H., M.L., and J.Z. took responsibility for the experiments; Y.Z., M.J., and W.L. drafted the manuscript; Y.Z., Y.X., Y.S., and N.L. performed the in vivo experiments and data analysis; T.S., F.L., and J.L. provided scientific and technical supports; D.M., J.Y., and C.J. critically revised the manuscript. The manuscript was written through the contributions of all authors. All authors have given approval for the final version of the manuscript.

## Supporting information

Supporting Information

## Data Availability

The data that support the findings of this study are available from the corresponding author upon reasonable request.
