# Supplementary material for: Silencing of IRF8 Mediated by m6A Modification Promotes the Progression of T‐Cell Acute Lymphoblastic Leukemia
Source: Adv Sci (Weinh). 2022 Dec 7;10(2):2201724. doi: 10.1002/advs.202201724 (PMC9839875; doi:10.1002/advs.202201724)
Supplement: Supplementary file 1 — Supporting Information [file ADVS-10-2201724-s001.pdf]

ADVANCED SCIENCE
Open Access

Supporting Information

for *Adv. Sci.*, DOI 10.1002/advs.202201724

Silencing of *IRF8* Mediated by m6A Modification Promotes the Progression of T-Cell Acute Lymphoblastic Leukemia

Ying Zhou, Min Ji, Yuan Xia, Xiaoyu Han, Mingying Li, Wei Li, Tao Sun, Jingru Zhang, Fei Lu, Yanping Sun, Na Liu, Jingxin Li, Daoxin Ma, Jingjing Ye and Chunyan Ji**

Supplementary methods

Cell proliferation assay

CCK8 kit (BestBio, China) was applied to assess cell proliferation. Cells were seeded in 96-well plates with 100 μL of culture medium and maintained in a humidified incubator at 37 °C. At each time point for the next 6 days, 10 μL CCK-8 was added to each well and incubated at 37°C for 3 h. Absorbance at 450 nm was measured by a Synergy H1 Hybrid Microplate Reader (BioTek, USA).

5-Ethynyl-2'-deoxyuridine (EdU) staining assay

EdU staining assay was performed using the iClick™ EdU Andy Fluro 647 Flow Cytometry Assay Kit (ABP Bioscience, USA) following the manufacturer's instructions. Briefly, cells were incubated with 10 μM EdU Andy Fluor 647 staining solution for 2 h under growth conditions. Then cells were fixed and permeabilized, and analyzed by flow cytometry.

Cell cycle assay

Cell cycle analysis was conducted using the cell cycle kit (BestBio, Shanghai, China) according to the manufacturer's specifications. In short, 1×10^7 cells were collected and fixed with 70% ice-cold ethanol at −20°C overnight. Then cells were resuspended with 0.04 mg ml^{-1} propidium iodide (PI) solution containing 0.2 mg ml^{-1} RNase A and incubated at 4℃ for 30 min. Cell cycle distribution was measured using a BD FACSAria III flow cytometer (BD Biosciences, USA), and data were analyzed with ModFit LT 5.0 software.

Colony formation assay

0.5mL semi-solid methylcellulose medium (MethoCult™ H4230, StemCell) was applied to culture 5×10^3 T-ALL cells in 24-well plates in a humidified incubator at 37 °C. About 10 days later, the number of colonies over 50 cells were calculated

under 200× magnification using a microscope (Invitrogen, USA).

Migration and invasion assay

The migration potential was measured using transwell inserts fitted with 8 μm-pore-size polycarbonate filters (Corning Costar, USA) in a 24 well plate. Cells were seeded in the upper compartment in 0.5% FBS culture medium while the medium in the lower compartment contained 20% FBS. 24 h later, contents in the lower compartments were harvested and counted. The ratio of migrated cells to the total number of cells seeded in the upper compartments was considered as the migration rate.

Furtherly, the invasive potential was measured using transwell inserts coated with Matrigel (Corning Costar, USA) on the upper compartment. Cells were seeded in the upper compartment in 0.5% FBS medium while the culture medium in the lower compartment contained 20% FBS. 48 h later, the remaining cells in the upper compartment were gently pipetted out. At the same time, the cells on the underside of the membrane that had penetrated the filter were 4% paraformaldehyde-fixed and stained with 0.2% crystal violet. Finally, the cells were observed and captured on an inverted microscope. Besides, the migrated cells in the lower chamber were also collected by centrifugation and counted by cell counter. The ratio of migrated cell number to the total number of cells seeded was considered as the invasion rate.

Real-time quantitative- polymerase chain reaction *(qRT-PCR)*

Total RNA was isolated by using TRIzol (Invitrogen). For mRNA expression, 500 ng RNA was reverse-transcripted into cDNA with a 5× Evo M-MLV RT Master Mix (AG, China) in a total reaction volume of 10 μL. RT-qPCR was performed by Roche Light Cycler 480 Ⅱ (Roche, Switzerland) with 1ul of cDNA, appropriate primers and 2× SYBR Green Pro Taq HS Premix (AG, China) according to manufacturer’s instructions. GAPDH was applied as an internal control. Appropriate primers are listed in Supplementary Table 2.

Western blot

Cells were washed and ruptured with M-PER^{TM} Mammalian Protein Extraction Reagent (Thermo Scientific, USA) containing protease inhibitor cocktail, and phosphatase inhibitor cocktail. The lysates were resolved in SDS-PAGE and transferred onto nitrocellulose membranes (Millipore, USA). Membranes were blocked with 5% skim milk for 2 hours, followed by overnight incubation at 4 °C with anti-IRF8, anti-PIK3R5, anti-p-AKT, anti-AKT, anti-p-MTOR, anti-P27, anti-CCR2 (CST, USA), anti-FTO (ABclonal, China), anti-GAPDH (Abways, China). After being washed for 30 min with Tris-buffered saline containing 0.1% Tween 20, membranes were incubated with appropriate secondary antibodies for one hour at room temperature. Blots are visualized with FluorChem E Chemiluminescent Western Blot Imaging System (Cell Biosciences, USA). The relative signal density of all blots was quantified by ImageJ software in all experiments.

Immunohistochemistry

For the immunohistochemical study, heat-induced epitope retrieval was applied for tissue sections before incubation with anti-IRF8, anti-PIK3R5, anti-Ki67 (Proteintech, USA), and anti-p-AKT (CST, USA) antibodies at 4℃ overnight. Then the sections were incubated with appropriate secondary antibodies conjugated to biotin-streptavidin horseradish peroxidase (HRP) for 30 min at room temperature and stained using 3,3′ -diaminobenzidine (DAB) reagent. The slides were observed and photographed under a fluorescence microscope (Invitrogen, USA), and immunostaining results were analyzed using ImageJ software.

RNA-sequence

Total RNA was extracted from IRF8 overexpressed Molt4 cells or control Molt4 cells, as well as FB23-2 treated or control Molt4 cells using TransZol Up Plus RNA Kit (Cat#ER501-01, Trans). After that, qualified total RNA was purified using RNase-Free DNase Set (QIAGEN, Germany), and RNAClean XP Kit (Beckman Coulter, USA) following the manufacturer’s instructions. VAHTS Universal V6

RNA-seq Library Prep Kit (Vazyme, China) was used to prepare the libraries. Illumina NovaSeq6000 was used to conduct sequence in each group in triplicate (Biotechnology Corporation, China). Sequence reads were aligned to the human genome version 38 (hg38) following the standard analysis pipeline for the Illumina sequence.

Dot blot analysis

Total RNA was extracted with TRIzol (Invitrogen) and quantified by spectrophotometry (Denovix). Then 2 μL RNA sample was dropped on the front of the positively-charged Nylon transfer membrane (RPN303B, GE Healthcare) and UV crosslinked twice to the Nylon transfer membrane after dried. Later, blockage of the membrane was performed with 5% skim milk, followed by overnight incubation with the anti-m6A antibody (Synaptic Systems, Germany) at 4 °C. After 1h-incubation with the corresponding second antibody, the membrane was developed and visualized with FluorChem E Chemiluminescent Western Blot Imaging System (Cell Biosciences, USA). Semi-quantitative analysis of blot density was conducted using ImageJ software.

Analysis of m6A/A ratio using liquid chromatography-tandem mass spectrometry (LC-MS/MS)

Poly(A) mRNA was isolated from FB23-2 treated Molt4 cells and control cells. 8 μL mRNA was added into an 8μL mixture containing Tris-HCL, benzonase, phosphodiesterase Ⅰ, alkaline phosphatase, and RNase-free water. The mixture solution was then incubated at 37 ℃ for 5 h. After that, the solution was diluted 6 times to obtain the gradient concentration, and 10 μL of the diluted solution was loaded for measurement using Waters Acquity UPLC and AB SCIEX 5500 QQQ -MS. For the quantification of the m6A modified nucleosides, the nucleosides to base ion mass transitions of 282.1 to 150.1 Da were accessed under the MRM positive ESI mode. Data were analyzed using MultiQuant.

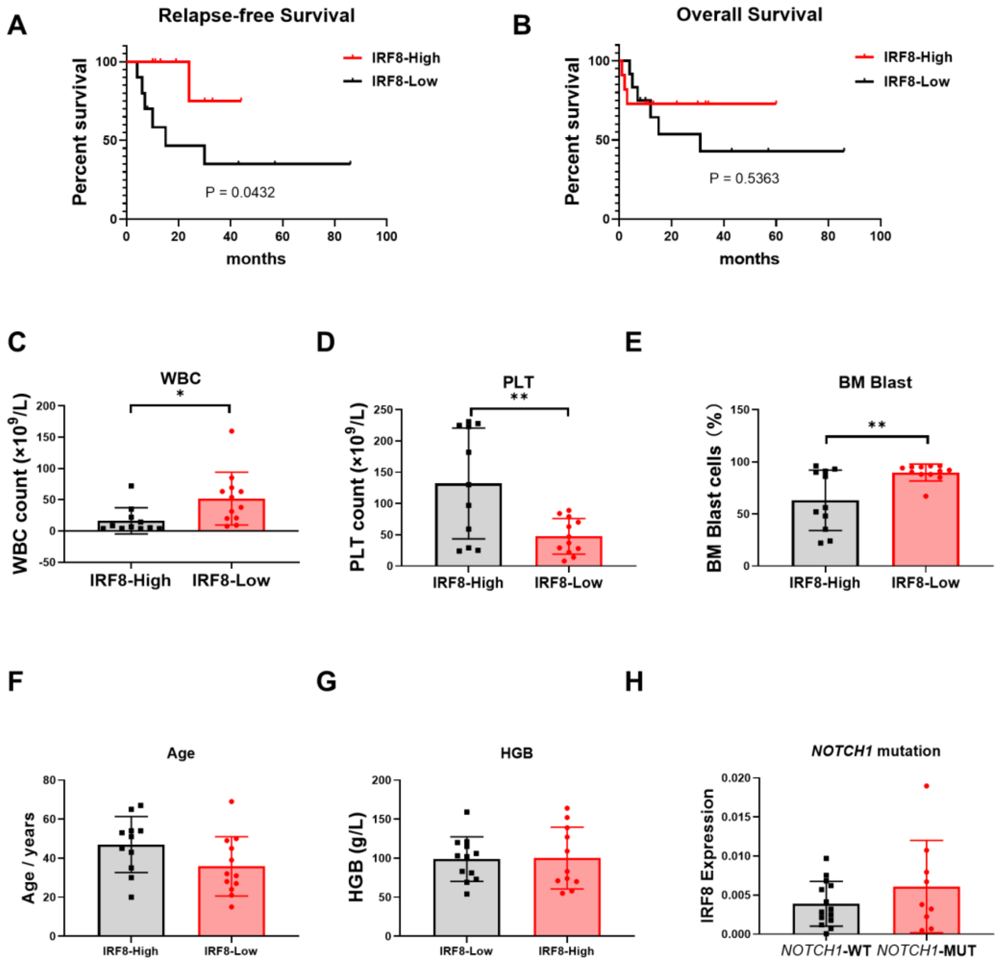

Supplementary Figure 1. Correlation between IRF8 levels and the prognosis of T-ALL patients. (A) Kaplan-Meier survival curve of relapse-free survival (RFS) and (B) overall survival (OS) of T-ALL patients grouped by IRF8-High (n = 11) and IRF8-Low (n = 12), analyzed by log-rank test. (C) The WBC counts, (D) PLT counts, (E) BM blasts, (F) age, (G) HGB values of T-ALL patients grouped by IRF8-High and IRF8-Low, analyzed by unpaired t-test. (H) IRF8 levels in T-ALL patients with wildtype *NOTCH1* (*NOTCH1*-WT, n = 14) and mutant *NOTCH1* (*NOTCH1*-MUT, n = 9). Unpaired, two-tailed Student's test, mean with SD, *P < 0.05, **P < 0.01.

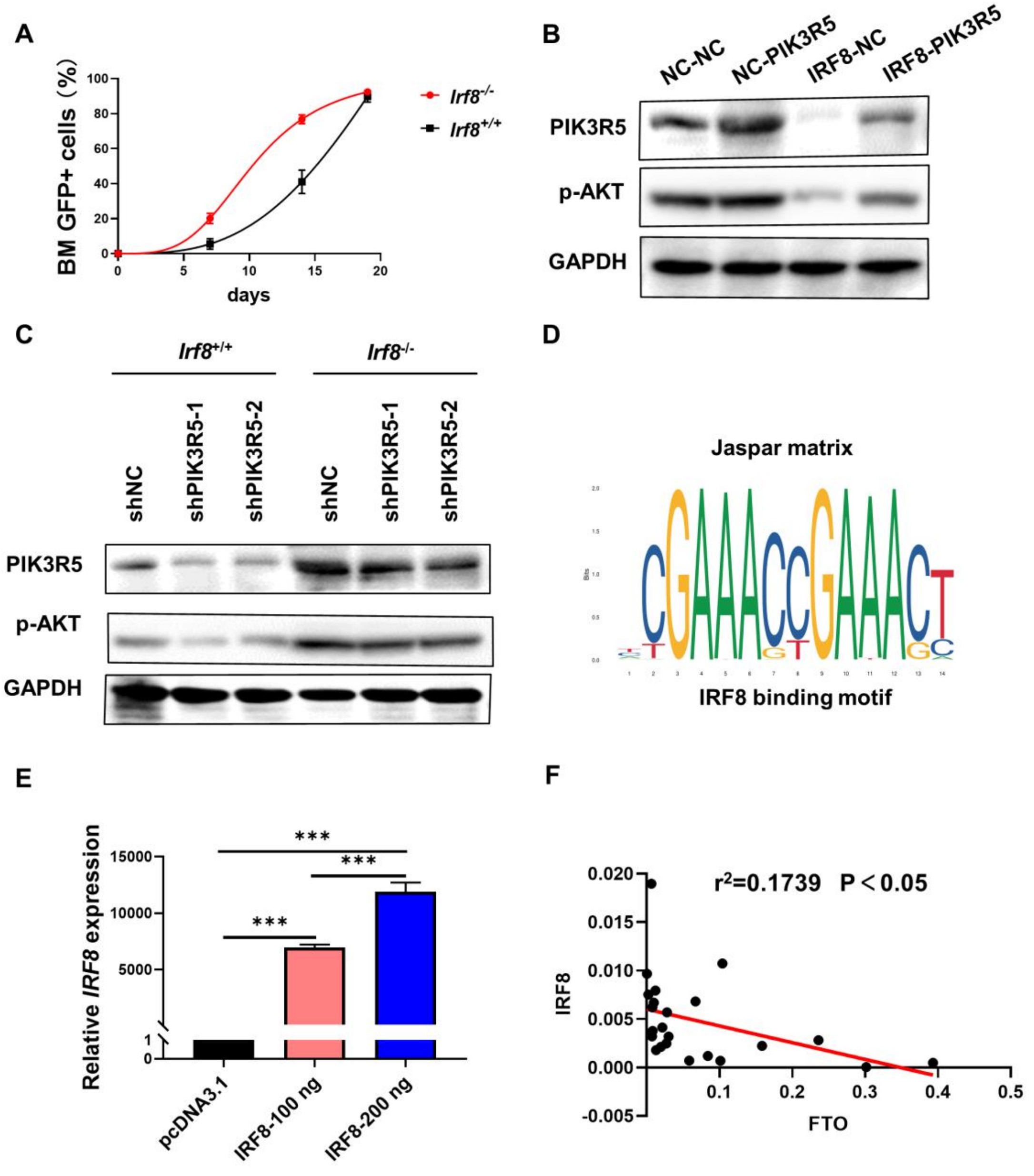

Supplementary Figure 2. (A) The proportions of BM GFP^{+} cell in *Irf8*$^{-/-}$ and *Irf8*$^{+/+}$ T-ALL mice with the elapse of time. (B) Immunoblot analysis of PIK3R5 and p-AKT levels in Molt4-IRF8 cells and Molt4-NC cells overexpressing *PIK3R5* or negative controls (NC). (C) Immunoblot analysis of PIK3R5 and p-AKT levels in BM cells from *Irf8*$^{+/+}$ *or Irf8*$^{-/-}$ T-ALL mice with or without PIK3R5 knockdown. (D) Potential motif in the promoter region of *PIK3R5* that can bind with IRF8 as predicted by Jaspar database. (E) qRT-PCR analysis of *IRF8* expression level in HEK293T cells transfected with empty vector, or *IRF8*-pcDNA 3.1 (100 ng or 200 ng). Analyzed by

One-way ANOVA. (F) Correlation between FTO and IRF8 expression across the 23 T-ALL samples by Pearson correlation analysis. *** P < 0.001.

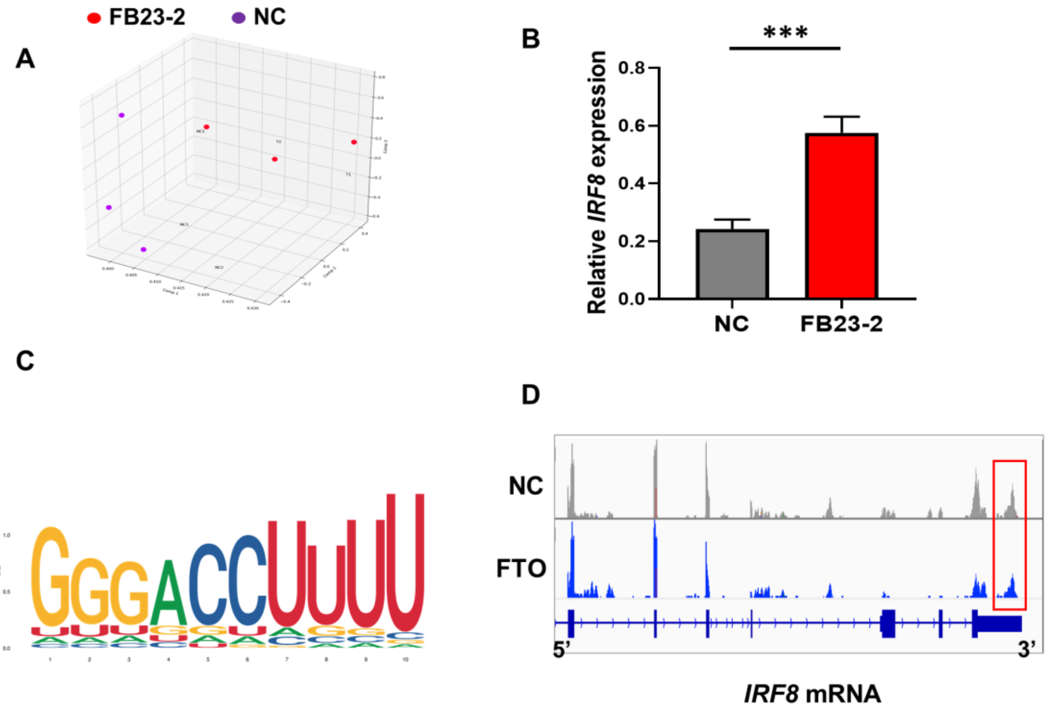

Supplementary Figure 3. (A) Principal component analysis (PCA) of gene expression characteristics in Molt4 cells incubated with 2 μM FB23-2 or DMSO (NC) for 48 h by RNA-seq (n = 3). (B) Relative expression of IRF8 in Molt4 cells incubated with FB23-2 at the concentration of 2 μM or DMSO (NC) for 48 h. Unpaired t-test. (C) Enrichment of the m6A motif RRACH (R = G or A; H = A, C or U) in Molt4 cells (P = 1e-48). (D) Analysis of the m6A abundance in *IRF8* mRNA transcripts in FTO-overexpressed leukemic cells and negative control (NC) cells. Data are downloaded from GSE76414. *** P < 0.001.

Supplementary Tables

Supplementary Table 1. Clinical characteristics of T-ALL patients and healthy donors.

**Characteristics**	**T-ALL patients (n=23)**	**Healthy donors (n=12)**
Gender		
Male	13	6
Female	10	6
Age median (range)/ years	43 (15-67)	42 (18-67)
WBC mean (range)/ $\times 10^9$/L	34.81 (3.79-159.68)	
HGB mean (range)/ g/L	99.45 (54-164)	
PLT mean (range)/ $\times 10^9$/L)	87.96 (8-231)	
BM blast cells mean (range) / %	76.97 (22-96)	
NOTCH1 mutation		
Positive	9 (39.13%)	
Negative	14 (60.87%)	

Supplementary Table 2. Sequences of qPCR primers and shRNAs used in the study.

Assays	Sequence	
**qRT-PCR**		
IRF8	Forward (5' to 3')	AGTAGCATGTATCCAGGACTGAT
	Reverse (5' to 3')	CACAGCGTAACCTCGTCTTC
FTO	Forward (5' to 3')	AGCATGGCTGCTTATTTCGG
	Reverse (5' to 3')	TTGTAGGTGCAGCCTGGATT
PIK3R5	Forward (5' to 3')	ACGTCTAACGATGGCTGGAAC
	Reverse (5' to 3')	CCACAAGGTCATGGTCATACG
GAPDH	Forward (5' to 3')	GGAGCGAGATCCCTCCAAAAT
	Reverse (5' to 3')	GGCTGTTGTCATACTTCTCATGG
**MeRIP / RIP**		
IRF8-IP1	Forward (5' to 3')	GTGATGGCCTGGATGCTGTAA
	Reverse (5' to 3')	CAAATTCCTTTATCGAAGCAGCTA
IRF8-IP2	Forward (5' to 3')	GCTGCTTCGATAAAGGAATTTGGA
	Reverse (5' to 3')	TCTTGTACTCATTAAAGGGGGCT
IRF8-IP3	Forward (5' to 3')	GTGACTGTGAGAGTTTCCGGT
	Reverse (5' to 3')	TCCCCTGGTCCTCTCCTCTA
IRF8-IP4	Forward (5' to 3')	CGTGGCTTGTCCCTTTTGTC
	Reverse (5' to 3')	TGAATTGGAGATTCAAGCCTATTGT
**CHIP-qPCR**		
PIK3R5	Forward (5' to 3')	CAGGCCTCTGTGTTCAACCAA
	Reverse (5' to 3')	TCTGTTAGAGGCGTGCAAACT
**shRNA**		
shIRF8-1	Sense (5' to 3')	CCAUACAAAGUUUACCGAATT
	Antisense (5' to 3')	UUCGGUAAACUUUGUAUGGTT
shIRF8-2	Sense (5' to 3')	GAUUGACAGUAGCAUGUAUTT
	Antisense (5' to 3')	AUACAUGCUACUGUCAAUCTT

shFTO-1	Sense (5' to 3')	GCAGCAUACAACGUAACUUTT
	Antisense (5' to 3')	AAGUUACGUUGUAUGCUGCTT
shFTO-2	Sense (5' to 3')	GGAUGACUCUCAUCUCGAATT
	Antisense (5' to 3')	UUCGAGAUGAGAGUCAUCCTT
shPIK3R5-1	Sense (5' to 3')	GCCTGTGAAGCGAAGTCATGGTT
	Antisense (5' to 3')	CCAUGACUUCGCUUCACAGGCTT
shPIK3R5-2	Sense (5' to 3')	GCAATGTACTGGGCCTCATGCTT
	Antisense (5' to 3')	GCAUGAGGCCCAGUACAUUGCTT
NC	Sense (5' to 3')	UUCUCCGAACGUGUCACGUTT
	Antisense (5' to 3')	ACGUGACACGUUCGGAGAATT
